# Construction and validity evidence of a scale for coping with difficult social academic interpersonal situations

**DOI:** 10.1186/s41155-018-0103-2

**Published:** 2018-09-03

**Authors:** Adriana Benevides Soares, Luciana Mourão, Fátima Almeida Maia, Humberto Claudio Passeri Medeiros, Marcia Cristina Monteiro, Roberta de Souza Nogueira Barros, Pedro Vítor Souza Rodrigues

**Affiliations:** 1 Universidade Salgado de Oliveira, Niteroi, Brazil; 2 Universidade do Estado do Rio de Janeiro, Rio de Janeiro, Brazil

**Keywords:** Coping, Factor analysis, Psychological evaluation

## Abstract

The aim of this study was to develop a scale to investigate the ways of coping with interpersonal situations considered difficult in the Brazilian university context. The items were based on the results of a study previously obtained in a focus group with university students. The first steps of the study were the analysis of judges and the investigation of the semantic validity. After these steps, a total of 1366 (female = 74.9%) students from public and private institutions participated in the study. The study followed the steps design of the items, development of the first version of the instrument, and initial tests of validity (content validity and internal consistency). The results of the exploratory factor analysis indicated the maintenance of 26 items, distributed in four factors: focus on emotion (*α* = 0.73), focus on social support (*α* = 0.81), focus on religious coping (*α* = 0.83), and focus on the problem (*α* = 0.70). The final scale solution was considered satisfactory for the proposed instrument, with consistency for the application in other studies and investigations that evaluate the coping strategies of students in situations considered difficult in the university.

## Background

Higher education in Brazil has undergone considerable changes in the last 20 years. Some of these changes have involved the opening of new institutions and the amplification of student places. However, this growth has not been accompanied by measures to guarantee the continuance of the student or the quality of the interpersonal relationships, of the learning, and of the conditions that encourage the student to deal with the challenges of university admission (Soares, Leme, Nogueira, Maia, & Lima, 2016b).

In view of the issues raised, starting university can be seen as a vulnerable moment in life, since the transition from one way of teaching to another can cause the students to experience distress (Bejerano, 2014; Fagundes, 2012; Oliveira, Carlotto, Vasconcelos, & Dias, 2014; Soares et al., 2014). Many students enter the university as adolescents, in a period in which the young person is undergoing various changes, involving personal and biological aspects, requirement for academic development, and adaptation to more autonomy in the sociocultural context, as well as cognitive aspects and gender roles (Awang, Kutty, & Ahmad, 2014; Neves & Pinheiro, 2009).

Changes in this stage of life are usually accompanied by a high level of stress, as the young person does not yet have many previous experiences to cope with the different challenges encountered in academic life (Soares & Del Prette, 2013). Among these challenges are the adjustment to new rules and requirements, the development of autonomy in relation to learning processes, and the construction of new relationships with peers, teachers, and supervisors.

Specifically, in relation to interpersonal situations, reports from university students indicate difficulties in student-student, student-teacher, and student-educational institution relationships (Soares, Gomes, Maia, Gomes, & Monteiro, 2016a; Soares et al., 2016a, 2016b). Situations perceived as difficult in the student-student relationships include the lack of commitment of the students to the course, the behavior of the colleagues perceived as undesirable during classes, individualism, and “cliques.” Changes in the behavior of the teacher during tests, the inadequate teaching method, the lack of empathy with the teacher, and arrogance of the teacher can be highlighted in the student-teacher relationships. Finally, difficulties associated with the student-institution relationships include a lack of information in situations such as room changes, class cancelations, and scientific events, as well as the lack of books in the library.

In this sense, in the day-to-day life of the university, the student deals with situations that are difficult to manage, such as requesting changes in the behavior of colleagues, rejecting abusive requests, giving and receiving criticism from peers and teachers, and expressing and defending opinions in public. These challenges, configured by the university context, can be very stressful, to the point of generating consequences that are harmful to the health of the student. High stress levels in university students are characterized by “emotional exhaustion” due to academic demands; “disbelief,” as an attitude distanced from the studies and “professional inefficiency,” identified through self-perceived incompetence (Borges & Carlotto, 2004).

In addition to stressful challenges, a high frequency of social anxiety conditions can be developed in this context, as demonstrated by the results highlighted by Neufeld, Godoi, Palma, and Crippa (2016), from the application of a social phobia inventory with university students. Their study demonstrated high percentages (over 30%) of students that indicated, for example, the difficulties of speaking to audiences or dealing with criticism. Appropriate coping strategies could prevent the development of these conditions by reducing stressors in the environment (Schaufeli, Martinez, Pinto, Salanova, & Bakker, 2002).

Sanzovo and Coelho (2007) also stated that learning coping skills is very important as this allows people to develop the capacity to deal with adverse, difficult-to-manage contingencies using a more adequate repertoire. Costa and Leal (2006), in turn, stated that the challenges presented to individuals require changes that facilitate adaptation to the context, with the quality of this adaptation being fundamental for mental health. In this sense, coping can be an important strategy for students and teachers (Araújo et al., 2016) in different stages of the academic life, from the beginning of elementary education, with positive results for persistence in challenging tasks and learning (Skinner, Pitzer, & Steele, 2016). The use of coping strategies can also help students to adapt to the university environment and their future careers (Murray, 2016).

Coping can be defined as the cognitive and behavioral efforts performed by individuals to fulfill demands and overcome difficulties created by their internal and external world, using this to control and reduce tensions (Folkman & Lazarus, 1985). It should be noted that the effect of coping with the context provides the criterion to evaluate the adaptability of the strategy, since a specific coping action can be adjusted to certain situations (Vinay, Esparbès-Pistre, & Tap, 2000). Personal characteristics, such as social environment, values, goals, and beliefs, may influence the adoption of forms of coping by individuals (Folkman & Lazarus, 1985).

According to Seidl, Tróccoli, and Zannon (2001), coping can be classified as being focused on the problem, emotion, social support, or religious practices/fantasy thinking. The first is understood as an effort to modify the situation that causes the stress; the second, as the effort to regulate the emotional state; the third concerns support from others; and the latter refers to behavior and thoughts based on religious beliefs to cope with adversity. In this sense, coping strategies focused on the problem, emotion, social support, and religious practices/fantasy thinking are the interest for the present study given that Brazilian studies point to their use by university students. However, these studies are still incipient (Carlotto, Teixeira, & Dias, 2015; Oliveira et al., 2014).

Several studies have been carried out to construct and validate instruments to evaluate coping strategies; however, none were identified for interpersonal situations considered difficult to manage by students. Vera-Noriega, Albuquerque, Alvarez, and Pimentel (2003) conducted a 600-person study to validate an adjusted version of the Mexican Coping Styles Scale (MCSS): the Coping Styles Scale (CSS) of Góngora and Reyes (1998), from the model validated for northwest Mexico by Vera-Noriega and Silva (2000). Three factors were evidenced: direct, revalorative-social, and evasive and emotional for six problem situations: life, school/work, friends, family, partner, and health. The factors define the three coping styles. The result showed that the direct style was more frequent among women, adolescents, the urban population, people with incomes of up to three minimum wages, those who had attended the second phase of elementary school, and among those of the Evangelical religion.

In another study on the construction of coping scales, Balbinotti, Barbosa, and Wiethaeuper (2006) aimed to verify the internal and factor consistency of the Multifactorial Coping Inventory for Adolescents (MCIA-43), with a sample of 285 elementary and high school students, aged 13 and 18 years of both genders. The Cronbach’s alpha values found were considered satisfactory, having obtained variations from 0.71 to 0.89; however, the measure is specific for adolescents. Sandín and Chorot (2003), in turn, conducted a study of the development and preliminary validity evidence of the *Cuestionario de Afrontamiento del Estrés* (CAE) with a sample of university students. The CAE aims to evaluate seven basic coping styles: (i) focused on problem solving, (ii) negative self-focus, (iii) positive re-evaluation, (iv) open emotional expression, (v) avoidance, (vi) seeking social support, and (vii) religion. The results showed that the Cronbach’s reliability coefficients ranged from 0.64 to 0.92. The analysis of the study also suggested that the CAE has a factor structure of seven basic dimensions of coping, highlighting evidence of empirical validity for the instrument and coping as a predictor of the influence of stress on health. This was the only measure found for university students. However, an adaptation of this scale would not fulfill the objectives of the study, since it was focused on coping with stress in general, while the aim of the study was related to coping with difficult situations in the social-academic dimension.

Another relevant study was conducted by Seidl et al. (2001). The authors investigated the factor structure of the MCSS, in the version adapted to the Brazilian population by Gimenes and Queiroz (1997), to measure coping strategies in relation to specific stressors. The sample consisted of 409 adults, and the strategies extracted were focused on the problem, emotion, religious practice/fantasy thinking, and seeking social support. The results suggested possibilities of application for research and professional and clinical interventions.

The review of the cited measures shows that the multidimensionality associated with coping strategies requires studies in this field to be well delineated, with psychometric qualities for conducting studies that lead to the correct identification of the phenomenon. Many instruments used in Brazilian studies are used for general populations and are not specific to university students (Balbinotti et al., 2006; Costa & Polak, 2009; Neves & Pinheiro, 2009). Furthermore, results of focus group studies and a review of the literature (Soares et al., 2016a, 2016b) suggested there were five common interpersonal stressors in this situation: presentation at a seminar; receiving or giving criticism; refusing abusive requests; negotiating with peers, managers, or teachers; and joining a new class or group. Therefore, the purpose of this study is to assess four ways of coping with the five stressors. For this, a scale for Brazilian university students related to coping with interpersonal situations considered difficult previously reported in the literature (Soares & Del Prette, 2013; Soares et al., 2014; Carlotto, Teixeira, & Dias, 2015) was constructed.

## Methods

### First step: bibliographic search

The bibliographic search for difficult interpersonal situations was based the performance of focus groups with university students (Soares et al., 2016a, 2016b). The statements obtained in the focus groups were compared with the literature of the area to support the creation of the items. An investigation of articles on the subject of stress in university life was also carried out in order to identify coping strategies for stressful situations (Bardagi & Hutz, 2011; Deasy, Coughlan, Pironom, Jourdan, & Mannix-McNamara, 2015; Ito & Matsushima, 2016; Sanzovo & Coelho, 2007; Soares & Del Prette, 2013; Teixeira, Dias, Wottrich, & Oliveira, 2008; Woyciekoski, Natividade, & Hutz, 2014). The coping strategies were frequently related to the health area (Bardagi & Hutz, 2009; Deasy et al., 2015; Moraes, Koller, & Raffaelli, 2012; Sanzovo & Coelho, 2007; Teixeira et al., 2008; Tobin, 2004), and no studies were found that associated interpersonal situations in the university with coping strategies.

### Second step: definition of the parameters

Based on the coping concept (Antoniazzi, Dell’Aglio, & Bandeira, 1998; Folkman & Lazarus, 1985), two parameters were defined for the construction of the instrument: the interpersonal situations considered difficult in the academic context (described in this section) and the coping strategies (defined in the third step). The situations considered difficult by higher education students were listed, based on the work of Soares and Del Prette (2013) and articles referring to the use of focus groups in qualitative research (Soares et al., 2016a, 2016b). Initially, 12 situations considered difficult to manage were selected. These situations involved difficulties in exposing themselves publicly (presentation of work, when making questions, or making requests to the class); receiving or giving criticism; refusing abusive requests; negotiating with peers, managers, or teachers; and joining a new class or group. The 12 initial situations involving the use of assertiveness were grouped into five sets, identifying different assertive behaviors according to the literature (Soares & Del Prette, 2013; Soares et al., 2016a, 2016b): dealing with criticism, making claims, expressing themselves publicly in academic activities or giving ideas and opinions, and knowing how to refuse inconvenient requests, as shown in Table 1.

**Table 1:** Characterization of the five difficult coping situations

Situations	Description
Presentation at a seminar	Analysis of the difficulty in facing a classroom presentation during the presentation of work.
Not allowing a colleague who does not work to join the group	Moment when a colleague, already known for not helping in the performance of the work, asks to be integrated into the group. Difficulties in denying this inclusion.
Expressing opinion on the teaching method	Intervention in situations where the student does not agree with the way the teacher develops the content of the discipline.
Receiving academic criticism from colleagues	The way different judgments from colleagues regarding the academic conduct are dealt with.
New in class	Coping with “breaking the ice” in a new group.

### Third step: design of the items, definition of the response scale, and identification items

From the definition of the five interpersonal situations, four categories of evaluation were constructed that corresponded to possible coping strategies, as shown in Table 2. For each interpersonal situation, four to six items were designed for each category, which corresponded to possible reactions of the students in the attempt to cope with the situation. These were constructed so that strategies adjusted to the good performance of each activity were considered. Thus, in the first part of the item, the problem situation was presented (defined in the second step) and in the second part the possible coping strategies associated with it. A review was made regarding the clarity and relevance of the items in the day-to-day academic life.

**Table 2:** Definition of coping strategies

Category	Description
Coping strategy focused on the problem	Represents behavior of approaching the stressor in order to solve the problem, deal with, or manage the stressful situation. Includes behaviors that involve active efforts aimed at reassessing the problem, or restructuring it by perceiving it in a positive way.
Coping strategy focused on social support	Refers to seeking instrumental or informational support with strategies to cope with the stress-causing situation.
Coping strategy focused on emotion	Represents the effort to regulate the emotional state that is associated with stress or is the result of stressful events. This effort is directed at a somatic level or at a level of feelings aiming to alter the emotional state of the student.
Coping strategy focused on “powerful others”	Making use of religious or superstitious beliefs and behaviors to facilitate problem solving and to prevent or alleviate the negative emotional consequences of stressful life circumstances.

The response alternatives were arranged in a Likert type scale of five points: (1) never, (2) rarely, (3) sometimes, (4) almost always, and (5) always. This scale was chosen because it is one of the most used and recognized and because it is easy to understand, since it presents points that indicate the level of agreement in relation to a series of statements referring to positive or negative responses to a psychological object (Pasquali, 2012).

The identification items were requested: gender, age, course grade, educational institution, course, study period, social class, and income coefficient. This information aimed for a better understanding of the results, from a detailed characterization of the sample of participants. Prior to the application of the instrument, the confidentiality of the sociodemographic information was guaranteed to the participants.

### Fourth step: design of the first version and semantic validity of the instrument

After a critical and rigorous analysis, the items were standardized with the same linguistic model to facilitate the reading of the students that responded to the questionnaire. At this time the questionnaire contained 100 items. A semantic validity process of the initial version was carried out with the support of university students who responded to the instrument and highlighted possible doubts or suggestions for improvements in the design of the items.

### Fifth step: content validity

The content validity was performed by judges who evaluated the indicators regarding clarity of the language, using a Likert scale from 1 to 5: “nothing,” “a little,” “average,” “very,” and “totally,” and regarding their suitability in relation to the structure of the theoretical model, which corresponded to the four types of coping strategies listed. This version of the instrument included the constitutive definitions of coping strategies, and the items were laid out in a grid composed of columns for evaluation according to the criteria cited. The analysis was carried out by five independent judges. The criterion of permanence of the items was that each presented a consensus of 80% of the five participating judges (all PhDs in Psychology.

### Sixth step: definitive study

A total of 1366 Brazilian university students, aged from 17 to 39 years (*M* = 24.65 and SD = 5.09), mostly from private institutions and single, participated in this study, with approximately 25% of the sample from the first year (1st and 2nd grades) and predominantly of the B2 social class, as shown in Table 3. The social class was calculated using the *Critério Brasil* (ABEP, 2015), which takes into account the education level of the head of the family, the existence of monthly domestic servants, and the possession of consumer goods.

**Table 3:** Characterization of the sample

	Number	Percent
Institutions		
Public	255	18.67
Private	1111	81.33
Marital status		
Single	1024	74.96
Married	279	20.43
Other	58	4.25
Did not answer	5	0.37
Gender		
Female	1.022	74,87
Male	343	25,13
Course grade		
1st	158	11.57
2nd	258	18.89
3rd	145	10.61
4th	179	13.10
5th	143	10.47
6th	219	16.03
7th	83	6.08
8th	114	8.35
From the 9th	67	4.90
Social class		
A	144	10.54
B1	217	15.89
B2	413	30.23
C1	346	25.33
C2	204	14.93
D/E	42	3.07

Prior to the start of the data collection, the research project was entered in the *Plataforma Brasil* and approved by a Research Ethics Committee (authorization No. 66060117600005289), with all ethical procedures for research with human subjects followed. A list of 70 items, distributed among the five interpersonal situations, associated with a scale of answers with four response possibilities (strategy focused on the problem, on the emotion, on religious coping, and on social support) was applied with the participants in their free time. The applications were, in most cases, performed with groups of 20 participants.

The Statistical Package for the Social Sciences—SPSS, version 21.0 was used. Initially, the data were subjected to exploratory and descriptive statistical analyzes. The occurrence of univariate extreme cases (outliers) was evaluated, from the transformation of the variables into standardized scores (*Z*). The criterion for the possible exclusion of cases was values equal to or greater than 3 (*p* < 0.001, two-tailed), with the subjects only being removed from the analyses for which they were outliers.

To obtain validity evidence for the scale, factor analyses were performed using the Principal Axis Factoring (PAF) method, with oblique rotation (direct oblimin), since the construct assumes correlations between the factors, which were confirmed after the extraction of the factors. For the definition of the number of factors, a combination of three criteria was used: (i) parallel analysis, based on a hypothetical set of correlation matrices of variables, using the same number of variables and subjects as the empirical study that was performed (number of random Pearson correlation matrices = 500); method to obtain random correlation matrices: permutation of the raw data; (ii) minimum average partial (MAP) test, based on the portion of the systematic and non-systematic variance remaining in a correlation matrix after an increasing factor extraction (Damásio, 2012); and (iii) theoretical consistency, evaluating the consistency of each of the dimensions suggested by the statistical criteria (Pasquali, 2012). Cronbach’s alpha coefficient was used to verify the reliability of the extracted factors.

## Results and discussion

According to judges’ evaluation, a total of 70 items were maintained in the scale. Initial exploratory analyses indicated that there were no very influential outliers and that the distribution of data approached a normal distribution, without the need to exclude extreme cases. The missing cases presented a random distribution and were less than 1%, so we opted to remove them one by one from the analyses. The factoriality of the data matrix was confirmed by the Kaiser-Meyer-Olkin—KMO indicator of 0.85. Bartlett’s sphericity test was also significant (*p* < 0.001, estimated in *χ*^2^(325) = 8476.2); however, this was already expected given the sample size. Considering that the data presented good factoriality conditions, the next step was to determine the number of factors to be extracted to identify an underlying structure in the data matrix and to determine the number of latent variables (dimensions) that represented the observed variables.

For the definition of the number of factors, initially, the eigenvalues​ and the Kaiser criterion were analyzed, indicating the possibility of up to five factors, with the screeplot being able to discriminate four. The criteria of parallel analysis and MAP (minimum average partial), which are more robust for indicating the number of factors (Damásio, 2012), confirmed that the best factor structure would be that of four factors (empirical eigenvalues presented in Table 3). The existence of a possible fifth factor was ruled out by parallel analysis, since the fifth empirical eigenvalue (1.11) was lower than the corresponding random eigenvalue (1.37).

The following factors were found: the first denominated focus on social support (*α* = 0.81) and was composed of eight items; the second focus on religious coping (*α* = 0.83), with five items; the third focus on the problem (*α* = 0.70), consisting of seven items; and finally, focus on emotion (*α* = 0.73), with six items. Extraction of these factors indicated the maintenance of 26 items, as presented in Table 4. Two criteria were used to maintain the items in the scale: (*i*) loadings above 0.32 (Hair, Black, Babin, & Anderson, 2010) and (*ii*) the items that loaded in two factors were maintained provided there was a difference equal to or greater than 0.30 between the factor loadings in each of the factors. Many items were considered confusing because they are measuring more than a unique dimension (they presented factorial load in more than one factor, with small differences (< 0.30) in the values of these factorial loads). Thus, our decision was to delete them, and the definitive version of the scale got 26 items. New tests of parallel analysis and MAP confirmed the existence of four factors in this scale version. The results shown in Table 4 consider the factorial analysis taking into account the 26 items distributed in the four factors.

**Table 4:** Factor analysis (Principal Axis Factoring) relating items and factors extracted

	F1	F2	F3	F4
When...				
I want to express my opinion about the teacher’s method, I ask for help from the coordination to talk to him.	*0.49*			
I want to refuse a request from a colleague who wants to join the group without doing the work, I ask the class representative to solve the problem for me.	*0.63*			
I want to refuse a request from a colleague who wants to join my group without doing the work, I ask the teacher to explain my refusal.	*0.71*			
I receive academic criticism from colleagues in the classroom, I ask the colleague who criticized me to support me.	*0.62*			
I am new to the class, I ask the most outgoing people to introduce me.	*0.65*			
I am new to the class, I ask the teacher to do an activity that facilitates my integration.	*0.61*			
I receive academic criticism from my colleagues in the classroom, I ask a friend to defend me.	*0.67*			
I am new to the class, I ask the teacher to introduce me.	*0.74*			
I want to express my opinion on the teacher’s method, I ask “God” for more understanding.	0.22	*0.69*		
I have to express myself during the presentation of a seminar, I ask “God” to help me carry it out.		*0.81*		
I receive academic criticism from classmates in the classroom, I pray to “God” to make me emotionally strong enough to listen to them.	0.28	*0.68*		
I have to express myself during the presentation of a seminar, I ask “God” not to make me go blank during the speech.		*0.79*		
I receive academic criticism from colleagues, I ask “God” for the wisdom to deal with the criticism intelligently.		*0.78*		
I want to express my opinion on the teacher’s method, I try to be clear.			*0.62*	
I want to express my opinion about the teacher’s method, I ask him for an alternative method.			*0.60*	
I want to express my opinion about the teacher’s method, I think about the best way to do it.	− 0.26		*0.64*	
I have to express myself during the presentation of a seminar, I prepare myself for possible doubts.	− 21		*0.57*	
I receive academic criticism from colleagues in the classroom, I check with the teacher for the best way to study the content.		0.21	*0.57*	
I want to refuse a request from a colleague who wants to join my group without doing the work, I explain my principles.			*0.51*	
I want to express my opinion on the teacher’s method, I ask for an explanation in other words.			*0.60*	
I am new to the class, I try to disguise my embarrassment.				*0.52*
I receive academic criticism from colleagues in the classroom, I try to contain my insecurity.				*0.57*
I am new to the class, I suppress anxiety.				*0.67*
I am new to the class, I try to contain my discomfort.				*0.71*
I want to refuse a request from a colleague who wants to join my group without doing the work, I control my anxiety.				*0.66*
I want to refuse a request from a colleague who wants to join my group without doing the work, I seek to alleviate my discomfort.			0.22	*0.63*
Empirical eigenvalues	4.78	3.34	2.01	1.72
Random eigenvalues	1.47	1.44	1.41	1.38
Explained variance (%)	18.4	12.9	8.0	6.6
Factor correlation: F1–F2 = 0.28*; F1–F3 = − 0.43*; F1–F4 = 0.19*; F2–F3 = 0.15*; F2–F4 = 0.33*; F3–F4 = 0.26*				

Note: Researchers interested in using this scale with Brazilian samples can request the Portuguese version from the authors

*p < 0.01

Considering the Hair et al. (2010) classification, Pearson’s correlation between the four factors indicated coefficients that can be classified as moderate (between the focus on the problem and focus on social support factors and between the focus on religious coping and the focus on emotion factors) and low (all other two factor combinations). This indicates the existence of different styles of coping with difficult situations in the academic environment and also a composition among these styles that indicates the pairing of focus on social support with focus on the problem and focus on emotion with focus on religious coping. In general, the results show that some of the students tended to focus on external solutions from religious coping, while others focused on problem solving, self-control, or social support. As these strategies represent distinct styles of dealing with difficulties in academic life (Seidl et al., 2001; Vinay et al., 2000), their identification can contribute to proposals on how to deal with each student profile, in order to support them in this period of adaptation to university life.

The adaptation of the student to the university implies changes, such as greater autonomy in the construction of interpersonal relations and in the adaptation to the requirements necessary for university performance. Students need to develop an active presence in the learning process, as well as in the various situations they face in the undergraduate course and in their own lives (Bejerano, 2014; Fagundes, 2012; Oliveira et al., 2014; Soares et al., 2014). In view of the difficulties encountered during the adjustment to higher education, it is necessary to investigate the coping strategies used by students, aiming to develop interventions that help in this moment of adaptation to the university (Murray, 2016; Schaufeli et al., 2002).

Exploratory factor analysis indicated adequate psychometric results for the instrument. The extracted factors, (1) focus on emotion, (2) focus on social support, (3) focus on religious coping, and (4) focus on the problem, maintained 26 original items of the scale, taking into account the criterion of validity in their factor loading. The factors of the scale achieved Cronbach’s alpha indices between reasonable and satisfactory, which means the instrument can be applied in coping studies with university students.

From the theoretical point of view, the scale can be considered consistent in measuring coping strategies, since the factors extracted present theoretical support (Antoniazzi et al., 1998; Dias, del Castillo Rodriguez, & López-Sánchez, 2015). Thus, the four factors found were adequate for the construct and corroborate the literature (Costa & Leal, 2006; Freire & Vera-Noriega, 2011; Oliveira et al., 2014; Soares et al., 2014).

The first factor, called focus on social support, refers to seeking help from peers, family members, or other members of the groups to which the individual belongs. The second, focus on emotion, concerns the ability to regulate the emotional state in order to cope with adverse situations that may generate subjective discomfort (Colossi, Calesso-Moreira, & Pizzinato, 2011). The third, called focus on the problem, factor refers to the cognitive and behavioral commitment to face situations that are difficult to manage (Seidl et al., 2001). Finally, the focus on religious coping refers to the perspective that the difficulties are resolved without the active participation of the student. The instrument, in relation to the others found (Oliveira et al., 2014; Folkman & Lazarus, 1985, adapted by Savóia, Santana, & Mejias, 1996; Vitaliano, Russo, Carr, Maiuro, & Becker, 1985, adapted by Gimenes & Queiroz, 1997), fills a gap in the measurement of difficult interpersonal situations in the university environment. The identification of these situations and the measurement of the coping strategies adopted by the students can contribute to more efficient actions from the student support services. In this sense, studies with this scale could be useful for facilitating the implementation of activities that bring freshmen and veterans together through an exchange of information and experiences, facilitating peer relationships, as well as relationships with teachers, which are constructed in the classroom dynamics.

## Conclusions

In conclusion, the aim of this study was to construct a scale for Brazilian university students that covers coping with interpersonal situations considered difficult. The results indicate the existence of four strategies and styles of coping, these being focus on emotion, on social support, on religious coping, and on the problem. The structure of the scale obtained satisfactory psychometric indices that indicate an adequate, consistent, and relevant instrument for measurement in studies that aim to identify ways of coping with difficult situations in the university context. The initial tests of the validity of the scale reinforced its value in describing the coping phenomenon, since the maintenance of the evaluated factors was observed, allowing the measurement of behaviors used in university life.

One of the contributions of this study is related to the possibility for educational institutions to better understand the universe of coping strategies used by the students and their possible consequences. Therefore, it is recommended that universities carry out intervention programs for the promotion of student development or student support services. Studies with the scale presented here would make it possible to characterize the coping strategies most used by the university population, helping students to adjust to situations considered difficult and also providing information for decision-making regarding institutional policies.

The fact that the sample was limited to the state of Rio de Janeiro and some university courses can be considered a limitation, as cultural or profile characteristics of the students of certain areas can influence the results obtained. Another limitation of the study refers to the fact that the majority of the participants were female, mainly due to the predominance of this public in the courses that were the target of the data collection. It is therefore suggested that other investigations are carried out with students from other higher education institutions in Brazil and other areas of knowledge, in order to analyze possible differences and compare the results. As the study was carried out with a majority of university students of private institutions, it is suggested that future studies compare coping results in participants of public and private networks in order to analyze possible differences between these groups. Furthermore, only initial tests of the validity of the scale were performed. Therefore, it is important for future studies to continue exploring the scale in order to evaluate its temporal stability and predictive convergence, and confirm the four factors structure through confirmatory factor analysis.
